# Feasibility analysis of the SICKLECHECK™ test kit for rapid screening of sickle cell disease at a County Referral Hospital in Kenya

**DOI:** 10.4102/ajlm.v14i1.2739

**Published:** 2025-07-29

**Authors:** Antony S. Katayi, Phidelis M. Marabi, Stanslaus K. Musyoki

**Affiliations:** 1 Department of Medical Laboratory, Faculty of Laboratory, Bungoma County Referral Hospital, Bungoma, Kenya; 2 Department of Medical Laboratory Sciences, School of Health Sciences, Kisii University, Kisii, Kenya; 3 Department of Medical Laboratory Sciences, School of Health Sciences, Southeastern Kenya University, Kitui Kenya

**Keywords:** sickle cell disease, rapid test, testing, accuracy, Kenya

## Abstract

**Background:**

The burden of sickle cell disease in Western Kenya is substantial; however, there is limited research on the effectiveness of rapid diagnostic tests for the condition.

**Objective:**

This study evaluated the feasibility of using the SICKLECHECK™ rapid test kit for detecting sickle cell disease at Bungoma County Referral Hospital, Kenya.

**Methods:**

A cross-sectional study was carried out between October 2023 and February 2024 and included both healthy children and children with a known haemoglobin phenotype. The SICKLECHECK™ rapid screening test was compared to Bio-Rad^™^ high-performance liquid chromatography, which served as the reference standard. Sensitivity, specificity, positive predictive value, negative predictive value, and overall accuracy were calculated using MedCalc^™^ statistical software.

**Results:**

The study involved 194 children (98 girls and 96 boys), aged between 10 weeks and 15 years, with haemoglobin profiles sickle cell negative (*n* = 78), sickle cell trait (*n* = 21), and sickle cell disease (*n* = 95). The SICKLECHECK™ test demonstrated sensitivity, specificity, negative predictive value, and accuracy exceeding 97%, with a positive predictive value of 94.18% for haemoglobin A. It also effectively distinguished between normal (sensitivity 97.44%, specificity 99.14%), carrier (sensitivity 90.48%, specificity 98.27%), and disease (sensitivity 98.95%, specificity 98.99%) phenotypes.

**Conclusion:**

Based on the findings in this study, SICKLECHECK^™^ could be a reliable point-of-care diagnostic tool for sickle cell disease. The encouragement of healthcare facilities, especially in resource-limited settings, to adopt the SICKLECHECK^™^ rapid test for routine screening and diagnosis of sickle cell disease is recommended.

**What this study adds:**

This study highlights the diagnostic reliability of the SICKLECHECK^™^ rapid test in accurately identifying and differentiating sickle cell disease, trait, and normal haemoglobin phenotypes, reinforcing its potential role in strengthening early diagnosis efforts in clinical settings.

## Introduction

Sickle cell disease (SCD) is a chronic, severely debilitating autosomal recessive disorder caused by a single-point mutation in the β-globin gene. Under hypoxic conditions, sickle haemoglobin undergoes polymerisation, leading to the reversible deformation of red blood cells into a sickle shape.^1^ Globally, SCD is recognised as a major public health concern, with an estimated 300 000 to 400 000 infants born annually with the disease.^2^ The burden is heaviest in low-resource settings, where access to early diagnosis and effective care remains limited.^3^

Sub-Saharan Africa accounts for approximately 75% of global SCD births,^4^ and studies report that 50% to 80% of affected children in the region die before the age of five.^2^ Screening of carriers in at-risk populations has been suggested since both parents as carriers carry a one in four likelihood of passing a homozygous offspring.^5^ The World Health Organization has recently called on governments and international organisations to implement strategies for precise documentation of morbidity and mortality. This effort aims to address haemoglobinopathies, which are becoming more prevalent in society, posing a growing challenge to healthcare systems.^6^ Although several advances have been made in molecular diagnostics, their applicability in resource-constrained settings remains limited.^7^ There is a growing body of literature emphasising the need for simplified, accessible, and scalable testing options of SCD, particularly in high-burden areas.^8^ There is a need for an affordable and efficient method for accurate and early detection, given the high prevalence of haemoglobin anomalies in malaria-endemic regions such as Kenya.^5^

In Kenya, SCD and other haemoglobinopathies are highly prevalent, particularly in malaria-endemic regions such as Western Kenya and the coastal areas.^5^ Recent data from Bungoma County show a sickle cell trait prevalence of 14.28% among blood donors.^9^ Although homozygous SCD poses a significant burden in Western Kenya, studies on the effectiveness of rapid testing for the condition are scarce. This study assessed the practicality of using the SICKLECHECK™ test for rapid SCD screening at Bungoma County Referral Hospital, Kenya. This study specifically examined the sensitivity, specificity, positive predictive value (PPV), negative predictive value (NPV), and accuracy of SICKLECHECK™ in comparison to Bio-Rad™ high-performance liquid chromatography (HPLC), the reference method.

## Methods

### Ethical considerations

The study received approval from the Jaramogi Oginga Odinga Teaching and Referral Hospital Institutional Scientific and Ethical Review Committee (JOOTRH – ISERC) (reference number ISERC/JOOTRH/742/23). Authorisation to conduct the research was granted by the National Commission for Science, Technology, and Innovation (reference number NACOSTI/P/23/29577). Permission for data collection was obtained from Bungoma County Referral Hospital (reference number BDH/S&E/11/1/[256]). The study’s objectives, procedures, potential risks, and benefits were clearly explained to both the participants and their parents or legal guardians. Written informed consent was obtained from the parents or legal guardians of all participants. Participation was entirely voluntary, and withdrawal from the study was permitted at any stage without penalty. To ensure data confidentiality, digital files were secured with password protection, while physical documents were stored in a locked and restricted-access location. No personally identifying information was collected or retained.

### Study area

The study took place at Bungoma County Referral Hospital, Bungoma, Kenya, from 11 October 2023 to 22 February 2024. Bungoma County Referral Hospital is a level six hospital situated in the administrative centre of Bungoma County, Western Kenya. It serves as the primary referral facility for the county and its surrounding areas, providing healthcare to a population of 1 670 570 within a 2206.9 km^2^ region.^10^ Each year, around 3000 children attend the paediatric clinic at Bungoma County Referral Hospital, with SCD affecting approximately 5% of them, as reported in the hospital’s records.^11^ Sickle cell disease accounted for 7.8% of all paediatric admissions and was ranked among the top three causes of hospitalisation at Bungoma County Referral Hospital in 2023.^11^

### Study design and population

This study followed an analytical cross-sectional design. Included were children who already had a known haemoglobin phenotype and were being monitored at the sickle cell clinic of Bungoma County Referral Hospital in Kenya, as well as children who were apparently healthy and visiting the maternal and child clinic of Bungoma County Referral Hospital in Kenya.

### Sample size determination and sampling technique

The sample size for the study was determined using Fisher’s exact test,^12^ based on a previously reported prevalence of 14.8%.^9^ Using this prevalence and standard parameters for statistical confidence and margin of error, the calculated sample size was approximately 194 participants, which was adopted for the study. A systematic random sampling method was employed, selecting every second child who met the inclusion criteria until the desired sample size was achieved.

### Data collection

#### Recruitment and consent

The positive cases consisted of children aged 10 weeks to 15 years attending the sickle cell clinic at Bungoma County Referral Hospital with a known diagnosis of SCD (HbSS phenotype, i.e., homozygous for sickle cell haemoglobin) or sickle cell trait (HbAS phenotype, i.e., heterozygous for sickle cell haemoglobin), confirmed by prior haemoglobin electrophoresis or HPLC results. Healthy children within the same age range attending the Child Welfare Clinic for routine vaccinations were systematically selected as controls and presumed to be negative for SCD. Inclusion criteria for both groups included age between 10 weeks and 15 years, availability of haemoglobin profile for positive cases, and provision of informed consent by a parent or guardian. Exclusion criteria included recent blood transfusion (within the last 3 months), current use of antibiotics or hydroxyurea, presence of acute illness, or lack of consent. Participants were categorised into two groups, namely children with known SCD and healthy children without the disease. Parents or guardians received a detailed information sheet explaining the study objectives, participant selection process, procedures, potential risks and benefits, confidentiality measures, voluntary nature of participation, data sharing, and contact details for study-related inquiries. Following parental consent, children were assigned a unique identification number and included in the sample collection process.

#### Sample collection

For determination of the sickle cell phenotype, blood samples were collected from each participant. Approximately 2 mL of whole blood was drawn into a vacuum collection tube containing ethylenediaminetetraacetic acid, gently inverted 8–9 times, and labelled with a unique participant identifier. The samples were promptly delivered to the laboratory. If not tested immediately, the samples were stored at 2 °C – 8 °C for up to 24 h. Prior to testing, stored samples were allowed to reach room temperature and were mixed thoroughly, according to the manufacturer’s guidelines. Laboratory results were recorded using the study’s standard data collection form.

### Laboratory analysis

The performance of the SICKLECHECK™ (Zephyr Biomedicals, a division of Tulip Diagnostics (P) Ltd., Goa, India) rapid test kit for detecting sickle cell phenotypes was evaluated in comparison to the Bio-Rad™ (Bio-Rad Laboratories, Marnes-la-Coquette, France) HPLC method.

#### Sickle cell phenotype testing using SICKLECHECK™

SICKLECHECK™ operates on the principle of antibody/antisera agglutination with the corresponding antigen in a competitive immune-chromatography format. It utilises nanogold particles as the agglutination detection agent. The conjugate pad contains two components: a monoclonal antibody for haemoglobin S (HbS) and a monoclonal antibody for haemoglobin A (HbA), each conjugated to colloidal gold.

The monoclonal antibody conjugated with colloidal gold, specific to either HbS or HbA, binds to the corresponding antigens in the test specimen. As the specimen flows through the membrane assembly by capillary action, the antibody–antigen complex moves along the membrane. In the absence of HbS and HbA on the membrane, the complex continues to the test regions (T1 and T2), where no band forms because of a lack of binding. The absence of colour bands at the test regions (T1 and T2) indicates the presence of the corresponding antigens (HbS or HbA), respectively, in the specimen.

In the presence of HbS and HbA, the monoclonal antibody–colloidal gold conjugate moves along the membrane through capillary action, where it is captured by the respective antigen coated on the membrane (T1 & T2), resulting in the formation of colour bands. The appearance of colour bands at the test regions (T1 and T2) indicates that the specimen does not contain the corresponding antigens (HbS or HbA). The membrane’s control region (C) is coated with goat anti-mouse immunoglobulin G (IgG), which causes any unbound colloidal gold conjugates to continue traveling along the membrane and become immobilised. The control band validates the test results and serves as a procedural control.

The components of the SICKLECHECK™ test kit were allowed to reach room temperature before use. The foil pouch was opened by tearing along the notch to remove the testing device. The desiccant pouch was checked to ensure it was blue; if colourless or pink, the test device was discarded and replaced. Once opened, the device was labelled with the participant’s identity and used immediately.

The device was placed on a flat surface, and the assay buffer vial was mixed and held upright to break off the tip. The buffer, which lyses red blood cells to release haemoglobin and ensures proper sample flow and reaction, was mixed with the specimen. Two drops of this mixture were dispensed into the specimen port (S). Visible coloured bands at test regions (T1 and T2) were observed over a 15-min period, depending on analyte concentration. Tests were not read or interpreted after 15 min.

Test results were interpreted as follows: a result was deemed invalid if no band appeared in the control region (C), regardless of the presence of bands in the test regions (T1 or T2). A normal result was indicated by coloured bands in both the control (C) and test region T1. The presence of bands in the control (C) and test region T2 suggested SCD or sickle cell in combination with other haemoglobinopathies. A band in the control region (C) with no bands in either test region (T1 or T2) was interpreted as sickle cell trait or its association with other haemoglobinopathies. The appearance of bands in the control (C), test region T1, and test region T2 indicated the presence of other haemoglobinopathies, thalassaemia, or a combination of haemoglobinopathies with thalassaemia. To ensure internal quality control, each test batch included positive and negative control samples with known HbA, HbS, and mixed phenotypes, which were tested alongside participant samples. Additionally, the laboratory participated in an external proficiency testing programme coordinated by a collaborating external quality assurance provider.

#### Sickle cell phenotype testing using high liquid performance chromatography

The Bio-Rad™ HPLC was used to separate and quantify haemoglobin variants in whole blood based on the cation exchange principle. To prepare the sample, 5 µL of ethylenediaminetetraacetic acid anticoagulated blood was mixed with 1 mL of haemolysis solution provided in the kit. The instrument was then loaded with the diluted samples, which were further processed with the appropriate haemolysing/wash buffer before being inserted into the analytical cartridge. A programmed buffer gradient with increasing ionic strength was pumped through the cartridge, separating haemoglobin fractions based on their ionic interactions with the cartridge material. These fractions passed through a flow cell, where absorbance was measured at 415 nm, with a secondary wavelength of 690 nm to eliminate background noise. The software generated a printed chromatogram showing all eluted haemoglobin fractions and their respective retention times. Peaks were attributed to specific ‘windows’ based on retention times, with each peak corresponding to a haemoglobin component. If an unknown peak was observed, it was identified by its retention time. Each analysis cycle took approximately 6.5 min, from sample loading to the final printed results. If the HbS peak was present without any detectable HbA, the patient was diagnosed with SCD (i.e., HbSS phenotype). In contrast, the presence of both HbA and HbS peaks, with HbA being predominant, indicated sickle cell trait (i.e., HbAS phenotype). Commercially prepared Bio-Rad™ control samples, containing known haemoglobin variant compositions, were analysed daily alongside participant samples. The laboratory participated in an established external quality assurance programme coordinated by an accredited external body. As part of this programme, blinded proficiency testing samples were periodically analysed, and the results were submitted for assessment against established consensus values.

#### Data quality assurance

A trained phlebotomist was responsible for collecting the blood samples, ensuring the correct volume was obtained to maintain data quality. The samples were processed and tested according to the standard operating procedures of Bungoma County Referral Hospital’s laboratory. Internal and external quality controls were consistently monitored and verified throughout the study. A second individual cross-checked the recorded results for accuracy. For enhanced validity and reliability, all statistical tests were performed using a Z-score of 1.96 and a 95% confidence level.

### Data analysis

Data were stored using Microsoft Excel (Microsoft Corporation, Redmond, Washington, United States), while MedCalc™ statistical software (MedCalc Software Ltd, Ostend, Belgium) was used to calculate the sensitivity, specificity, PPV, NPV, and accuracy of SICKLECHECK™ in comparison to Bio-Rad™ HPLC, which served as the reference method. Results were presented in tables. The software computed sensitivity by dividing true positives by the total of true positives and false negatives, specificity by dividing true negatives by the sum of true negatives and false positives, PPV by dividing true positives by the sum of true positives and false positives, and NPV by dividing true negatives by the sum of true negatives and false negatives. Accuracy was calculated by multiplying sensitivity by the sum of prevalence and specificity, then multiplying by (1 – prevalence).

## Results

### Patient characteristics

A total of 194 children were included in the analyses, comprising 90 boys (46%) and 104 girls (54%) (Table 1). Participants ranged in age from 10 weeks to 15 years. Of these, 95 (49%) were confirmed to have SCD (HbSS phenotype), 21 (11%) were carriers of the sickle cell trait (HbAS phenotype), and 78 (40%) were sickle cell negative (HbAA phenotype, i.e., homozygous for haemoglobin A). Among the HbSS group, 47 (49%) were boys and 48 (51%) were girls. Of the children with the HbAS trait, nine (43%) were boys and 12 (57%) were girls. Among HbAA participants, 34 (44%) were boys and 44 (56%) were girls.

**TABLE 1:** Basic demographic characteristics of the study population, Kenya, 11 October 2023 – 22 February 2024.

Participant characteristics	All phenotypes			Haemoglobin phenotype								
				HbSS			HbAS			HbAA		
	Range	*n*	%	Range	*n*	%	Range	*n*	%	Range	*n*	%
All participants	-	194	100	-	95	49	-	21	11	-	78	40
**Gender**												
Male	-	90	46	-	47	49	-	9	43	-	34	44
Female	-	104	54	-	48	51	-	12	57	-	44	56
Age	10 weeks – 15 years	-	-	5 months – 15 years	-	-	6 months – 14 years	-	-	10 weeks – 14 years	-	-

HbAA, haemoglobin phenotype homozygous for haemoglobin A (sickle cell negative); HbAS, haemoglobin phenotype heterozygous for sickle cell haemoglobin (sickle cell trait); HbSS, haemoglobin phenotype homozygous for sickle cell haemoglobin (sickle cell disease).

### Detection of phenotypes

The SICKLECHECK™ assay correctly identified 98 out of 99 children with HbA as true positives (99.0%), and 94 out of 95 children without HbA as true negatives (98.9%). There was one false positive (1.1%) and one false negative (1.0%) for HbA detection (Table 2). For HbS detection, the test accurately identified 115 out of 116 children with HbS as true positives (99.1%), and 76 out of 78 without HbS as true negatives (97.4%). Two false positives (2.6%) and one false negative (0.9%) were observed for HbS detection (Table 2). The SICKLECHECK™ accurately identified 76 out of 78 children with HbAA as true positives (97.4%), and 115 out of 117 children without HbAA as true negatives (98.3%). There was one false positive (0.9%) and two false negatives (2.6%) for HbAA detection (Table 3). For HbAS detection, the test correctly identified 19 out of 21 children as true positives (90.5%), and 170 out of 173 children as true negatives (98.3%). Three false positives (1.7%) and two false negatives (9.5%) were recorded for HbAS detection (Table 3). In detecting HbSS, the test identified 94 out of 95 children as true positives (98.9%), and 98 out of 99 as true negatives (99.0%). There was one false positive (1.0%) and one false negative (1.1%) for HbSS detection (Table 3).

**TABLE 2:** SICKLECHECK™ detection of haemoglobin A and haemoglobin S compared to Bio-Rad™ high-performance liquid chromatography, Kenya, 11 October 2023 – 22 February 2024.

Results	Bio-Rad™ HPLC		
	Positive	Negative	Total
**SICKLECHECK™ HbA**			
Postive	98†	1‡	99
Negative	1§	94¶	95
Total	99	95	194
**SICKLECHECK™ HbS**			
Positive	115†	2‡	117
Negative	1§	76¶	77
Total	116	78	194

HbA, haemoglobin A; HbS, haemoglobin S; HPLC, high-performance liquid chromatography.

† , TP, true positive;

‡ , FP, false positive;

§ , FN, false negative;

¶ , TN, true negative.

**TABLE 3:** SICKLECHECK™ detection of haemoglobin phenotypes AA, AS and SS compared to Bio-Rad™ high-performance liquid chromatography, Kenya, 11 October 2023 – 22 February 2024.

Result	Bio-Rad™ HPLC		
	Positive	Negative	Total
**SICKLECHECK™ HbAA**			
Postive	76†	1‡	77
Negative	2§	115¶	117
Total	78	116	194
**SICKLECHECK™ HbAS**			
Postive	19†	3‡	22
Negative	2§	170¶	172
Total	21	173	194
**SICKLECHECK™ HbSS**			
Postive	94†	1‡	95
Negative	1§	98¶	99
Total	95	99	194

HbAA, haemoglobin phenotype homozygous for haemoglobin A (sickle cell negative); HbAS, haemoglobin phenotype heterozygous for sickle cell haemoglobin (sickle cell trait); HbSS, haemoglobin phenotype homozygous for sickle cell haemoglobin (sickle cell disease). HPLC, high-performance liquid chromatography.

† , TP, true positive;

‡ , FP, false positive;

§ , FN, false negative;

¶ , TN, true negative.

### Efficacy of SICKLECHECK™

The SICKLECHECK™ assay showed a sensitivity of 97.98%, specificity of 98.95%, and overall accuracy of 98.80% for HbA detection (Table 4). The PPV for HbA was 94.18%, while the NPV was 99.65%. For HbS detection, sensitivity was 99.14%, specificity was 97.44%, and overall accuracy was 97.69%. The PPV for HbS was 89.04%, and the NPV was 99.85% (Table 4).

**TABLE 4:** Sensitivity and specificity of the SICKLECHECK™ assay for the detection of haemoglobin A and haemoglobin S, Kenya, 11 October 2023 – 22 February 2024.

Diagnostic assessment	Haemoglobin A		Haemoglobin S	
	Value (%)	95% CI	Value (%)	95% CI
Sensitivity (TP/[TP+FN])	97.98	92.89–99.75	99.14	95.29–99.98
Specificity (TN/[TN+FN])	98.95	94.27–99.97	97.44	91.04–99.69
PPV (TP/[TP+FP])	94.18	69.70–99.13	89.04	63.10–96.35
NPV (TN/[TN+FN])	99.65	98.62–99.91	99.85	98.93–99.98
Accuracy (sensitivity × prevalence + specificity × 1-prevalence)	99.80	96.07–99.82	97.69	94.45–99.30

Note: These values were dependent on disease prevalence of 14.28% in Bungoma County. The sensitivity, specificity, PPV, NPV and accuracy for HbA and HbS phenotypes were evaluated by the open Epi Info™ CDC statistical software.

CI, confidence interval; FP, false-positives; FN, false-negatives; TP, true-positives; TN, true-negatives; PPV, positive-predictive value; NPV, negative-predictive value.

SICKLECHECK™ showed a sensitivity of 97.44% for detecting HbAA, 90.47% for HbAS, and 98.98% for HbSS (Table 5). Specificity was 99.14% for HbAA, 98.27% for HbAS, and 98.99% for HbSS. The PPVs were 95.15% for HbAA, 90.06% for HbAS, and 94.45% for HbSS. Negative predictive values were 99.55% for HbAA, 98.34% for HbAS, and 99.82% for HbSS. The overall accuracy was 98.89% for HbAA, 97.11% for HbAS, and 98.98% for HbSS (Table 5).

**TABLE 5:** Sensitivity and specificity of the SICKLECHECK™ assay for the detection of haemoglobin HbAA, HbAS and HbSS, Kenya, 11 October 2023 – 22 February 2024.

Diagnostic assessment	Haemoglobin phenotype					
	HbAA		HbAS		HbSS	
	Value (%)	95% CI	Value (%)	95% CI	Value (%)	95% CI
Sensitivity (TP/[TP+FN])	97.44	91.04–99.69	90.48	69.62–98.83	98.95	94.27–99.97
Specificity (TN/[TN+FN])	99.14	95.29–99.98	98.27	95.02–99.64	98.99	94.50–99.97
PPV (TP/[TP+FP])	95.15	73.60–99.28	90.06	74.53–96.56	94.45	70.76–99.17
NPV (TN/[TN+FN])	99.55	98.27–99.89	98.34	94.08–99.55	99.82	98.72–99.97
Accuracy (sensitivity × prevalence + specificity × 1-prevalence)	98.89	96.20–99.85	97.11	93.67–98.98	98.98	96.35–99.88

Note: These values were dependent on disease prevalence of 14.28% in Bungoma County. The sensitivity, specificity, PPV, NPV and accuracy for HbAA, HbAS and HbSS phenotypes were evaluated by the open Epi Info™ CDC statistical software.

CI, confidence interval; FP, false-positives; FN, false-negatives; TP, true-positives; TN, true-negatives; PPV, positive-predictive value; NPV, negative-predictive value HbAA, haemoglobin phenotype homozygous for haemoglobin A (sickle cell negative); HbAS, haemoglobin phenotype heterozygous for sickle cell haemoglobin (sickle cell trait); HbSS, haemoglobin phenotype homozygous for sickle cell haemoglobin (sickle cell disease).

## Discussion

The current study demonstrated that the SICKLECHECK™ assay achieved over 97% sensitivity, specificity, NPV, and accuracy in detecting HbA and HbS variants, as well as all relevant haemoglobin phenotypes (normal, carrier, and disease). The findings are consistent with a study conducted in Ghana and the United States and published in 2019,^13^ which assessed the HemoTypeSC™ rapid testing method, reporting sensitivity and specificity above 99%, with an accuracy of 99.80% for detecting HbA and 97.69% for HbS. Similarly, the current results on the NPV for HbS agree with the same study,^13^ which documented a NPV of 99.8% for HbS. The present findings are also consistent with those of a study conducted in India and published in 2024,^14^ which evaluated a point-of-care rapid diagnostic test kit for screening SCD. The study reported the following performance metrics: for HbAA, a sensitivity of 98.04%, specificity of 99.35%, PPV of 99.50%, and NPV of 97.47%; for HbAS, a sensitivity of 99.07%, specificity of 98.81%, PPV of 97.25%, and NPV of 99.60%; and for HbSS, a sensitivity of 97.92%, specificity of 100%, PPV of 100%, and NPV of 99.60%. The current findings on accuracy are in agreement with a study conducted in the United States and published in 2016,^15^ that validated the diagnostic performance of the Sickle SCAN™ test, reporting an overall accuracy of 99% at the bedside. The current specificity results also align with those reported by a study conducted in Nigeria and published in 2019,^16^ which found that the HemoTypeSC™ test for sickle cell anaemia had a specificity of 99.9% under optimal field conditions. However, the current findings slightly differ in terms of sensitivity, which was somewhat lower at 93.4%. The current specificity findings also align with those of a study conducted in the United States and published in 2017,^17^ which developed and evaluated a competitive lateral flow assay, reporting a specificity of 100% for diagnosing sickle cell anaemia. However, while the current study reported a higher specificity, the United States study had a slightly lower specificity of 90.0%, indicating a modest difference in diagnostic accuracy. These findings also correspond with those of a study conducted at Cincinnati Children’s Hospital Medical Center, United States, and published in 2016,^18^ which evaluated the effectiveness of a rapid, point-of-care lateral flow immunoassay for diagnosing SCD. The study reported a sensitivity of 99.5% for HbS, and 98.3% for HbA. However, the current results show slight differences in specificity, which were 92.5% for HbS, and 94.0% for HbA. The present findings also contradict those of a study conducted in Haiti and published in 2019,^19^ which reported a sensitivity of 90.0% for Sickle SCAN™. The present findings also contradict those of a study conducted in Mali and published in 2024 on the comparative performance of two rapid diagnostic tests (Sickle SCAN™ and HemoTypeSC™).^20^ The study reported sensitivities of 96.10% and 95.22%, respectively, for detecting the HbAS genotype, and 81.67% and 78.33%, respectively, for detecting the HbSS genotype.

The conflicting findings reported in some studies may be attributed to factors such as the variable interpretation of faint test bands,^13^ suboptimal environmental conditions during field testing,^21^ and reduced specificity in neonates because of high levels of foetal haemoglobin, which can interfere with antigen–antibody interactions in certain immunoassays.^15^ These discrepancies highlight the importance of considering context-specific variables such as age, environmental testing conditions, and the regional prevalence of haemoglobin variants when assessing the performance of rapid diagnostic tests. Nevertheless, despite these occasional inconsistencies, the broader body of evidence, including the current study, strongly supports the effectiveness of lateral flow-based rapid diagnostics as reliable and scalable tools for screening SCD, particularly in low-resource settings.

While a few false positive and false negative results were noted in our study, such occurrences are not unusual and may arise from a combination of technical and biological factors. For instance, faint or borderline test bands in lateral flow immunoassays can complicate interpretation, potentially leading to misclassification.^13,22^ Additionally, biological variability in haemoglobin expression, such as elevated levels of foetal haemoglobin in neonates, the presence of rare haemoglobin variants, or coexisting haemoglobinopathies, may influence the assay’s ability to accurately detect specific haemoglobin types.^23^ These factors can affect antibody binding or visual detection, contributing to diagnostic discrepancies.^13^ Further investigation through larger, population-based studies is warranted to better understand and address these limitations.

The SICKLECHECK™ test demonstrates high sensitivity, specificity, and accuracy for detecting SCD and trait, comparable to other rapid point-of-care assays for SCD identification. Based on these results, the SICKLECHECK™ test is recommended as an effective rapid screening tool for haemoglobin variants and phenotypes.

SICKLECHECK™ offers significant benefits for testing in situations requiring minimal blood volumes. For example, it is particularly effective for testing infants who cannot provide sufficient blood through venipuncture, as it requires only 10 µL of blood from a finger or heel stick. Maintaining follow-up with patients after leaving healthcare facilities can be difficult, especially in rural areas. In many parts of rural Africa, including Kenya, haemoglobin screening is typically conducted at centralised hospitals or laboratories, necessitating the transfer of diagnostic specimens away from the point of care. This approach results in increased costs, logistical complexities, and delays in obtaining test results.^13^ Rapid point-of-care screening methods, such as SICKLECHECK™, effectively address these challenges. SICKLECHECK™ does not rely on instruments, electricity, or refrigeration, requires minimal training for operators, and provides results through visual inspection within 15 min. These features make it a cost-effective, fast, and accurate diagnostic tool in low-resource settings with high rates of SCD. With its substantial sensitivity and specificity, low sample volume requirements, independence from specialised equipment, ease of use, and rapid results, SICKLECHECK™ is a highly recommended tool for point-of-care screening of SCD and traits.

### Recommendations

The study recommends that SICKLECHECK™ be adopted as a rapid point-of-care test for SCD and trait because of its minimal sample requirement, lack of need for specialised equipment or electricity, ease of use, and quick result turnaround.

### Limitations

A limitation of our study was the inability to evaluate Haemoglobin C (HbC) detection using the SICKLECHECK™ test, as no participants with this variant were identified.

### Conclusion

Based on the findings in this study, SICKLECHECK™ could be a reliable point-of-care diagnostic tool for SCD.
